# Comparative Study on The Preventive Effect of Saffron Carotenoids, Crocin and Crocetin, in NMU-Induced Breast Cancer in Rats 

**DOI:** 10.22074/cellj.2016.3901

**Published:** 2016-12-21

**Authors:** Meysam Sajjadi, Zahra Bathaie

**Affiliations:** Department of Clinical Biochemistry, Faculty of Medical Sciences, Tarbiat Modares University, Tehran, Iran

**Keywords:** Chemoprevention, Initiation, Promotion, Tumor Volume, Latency Period

## Abstract

**Objective:**

Crocin (Cro) and crocetin (Crt) are two widely known saffron carotenoids, which exert anticancer effects by different mechanisms. Here, we investigated and compared the preventive effect of Cro and Crt at the initiation and promotion stages of breast cancer induction in an animal model.

**Materials and Methods:**

In this experimental study, female Wistar albino rats were injected with three doses of N-methyl-N-nitrosourea (NMU). The preventive intervention was done at different times for the initiation and promotion stages. Thus, Cro/Crt was administered by gavage 20 days before, or one week after, the first NMU injection, for the prevention at the initiation or promotion stages respectively. The treatment was repeated every three days, and continued up to the end of experiment. Tumor appearance was checked by palpation and some parameters were determined after sacrifice.

**Results:**

Tumor volume, latency period, and tumor number were significantly decreased in the rat groups treated with both saffron carotenoids for prevention at both the initiation and promotion stages. Tumor incidence was 77% due to NMU injection, which was decreased to 45 and 33% (on average) after Cro and Crt administration, respectively. In addition, enkephaline degrading aminopeptidase (EDA) was decreased significantly in the ovaries of the animals, however, changes in the brain were not significant.

**Conclusion:**

Crt/Cro showed a significant protective effect against the NMU-induced breast cancer in rats. However, Crt was more effective than Cro and prevention at the initiation stage was more effective than at the promotion stage.

## Introduction

Carcinogenesis has been conceptualized as a multistep process in which cells undergo different changes. The process can be divided into three different stages, including: initiation, promotion, and progression. Initiation occurs during the days immediately after uptake of, or exposure to, a carcinogen. After that, the carcinogen is distributed and transported to organs and tissues, where it binds covalently to target-cell DNA, leading to DNA-adduct formation. In this step irreversible DNA damage causes the transformation of exposed cell. The metabolic activation of some carcinogens and detoxification can also occur during this step. The second phase of carcinogenesis is promotion. This phase is characterized by multiple exposures to the carcinogen causing proliferation of the transformed cell to produce multiple cancer cells. The promotion stage is relatively lengthy. It starts approximately 1 week after carcinogen administration and may continue up to more than 10 years from the exposure. It is a reversible stage in which actively proliferating preneoplastic cells accumulate. Progression is defined for a description of all post-initiation events in neoplastic development. It is an irreversible process that involves the growth of a tumor with invasive and metastatic potential. In some types of cancer, duration of progression is less than one year (1-3).

Cancer prevention can be defined as any intervention at each of the above mentioned stages, and is defined as primary, secondary and tertiary prevention. In primary prevention the exposure is prevented or decreased; and/or the resistance of individuals to the exposure is increased. Secondary prevention is the early detection and treatment of disease. Tertiary prevention is the use of treatment and prevention procedures, as well as rehabilitation programs to improve the outcome of illness and prevent the recurrence of cancer among affected individuals. Chemopreventive phytochemicals can be used as an intervention in the carcinogenic process by blocking initiation or reversing the promotion stage of multistep carcinogenesis. They can also halt or retard the progression of precancerous cells into malignant phenotypes. As mentioned above, one of the aims of prevention is to stop progression (2, 3).

Breast cancer is the most frequent malignancy diagnosed in women worldwide. Animal models of cancer using various chemicals have been used for the investigation of the effectiveness of drugs and natural products for both chemopreventive and chemotherapeutic purposes (3-5). Among the chemicals used for breast cancer induction in rat, 1,2-dimethylbenz (α)-anthracene (DMBA) and N-nitroso-N-methylurea (NMU) are the most common (6-8). NMU is a highly specific carcinogen for the induction of tumors in the mammary gland with estrogen and progesterone receptors, which is very similar to human breast cancer; and in contrast to DMBA, does not require metabolic activation. NMU-induced mammary tumors are locally more aggressive than DMBA- induced tumors (3). We have recently shown that the expression of cyclin D1 and p21 were increased in NMU-induced breast cancer in rats (9). We have also shown that the increase in p21 is dependent on p53 expression (10).

The state and functioning of the breast depend on hormonal balance, and alteration of the hormonal balance can predispose to the development of breast diseases. As a consequence of cellular stress, many hormones and neurotransmitters may be released in the course of a neoplastic disease. Simultaneously, the antineoplastic defense mechanisms are activated. Enkephalins (Leu5-enkephalin and Met5-enkephalin, ENKs) are humoral neurotransmitters which can act in defense of the organism. ENKs may modify the endocrine functions of glands like the ovary, which are involved in steroid secretion. Both the ovary and thyroid functions are under the control of the hypothalamus–pituitary axis. ENKs act in the breast in different ways, such as modulating steroid receptors and protease secretion (11, 12). ENKs are inactivated after hydrolysis by specific enzymes named enkephalin-degrading tyrosyl aminopeptidases (EDA) (11), and the activity of this enzyme has been considered as a measure of ENKs.

Saffron (Crocus sativus L.) has been used, from ancient times, to treat various human diseases in different parts of the world. We have recently reviewed the most important aspects of saffron and its constituents as a cancer preventive or therapeutic plant products (13-15). Crocin (Cro), the main constituent of saffron, is responsible for its color and is the only water soluble carotenoid in nature. Cro is metabolized into crocetin (Crt). The therapeutic effects of both Cro and Crt against some types of cancer have been pointed out previously by us and other research groups (4, 5, 16-18). Cro and Crt act in a dose dependent manner, and it has been shown that Crt is more effective against gastric cancer (AGC) cells than Cro (18).

Further to our previous research in this field, here we investigate the preventive effect of Cro and Crt against NMU-induced breast cancer in female Wistar rats, at both the initiation and promotion stage.

## Materials and Methods

Eighty female Wistar rats were used in this experimental study. The animals, 30 days of age, were obtained from the animal house of Tarbiat Modares University. They were housed, two per cage, in a light-tight, environmentally controlled room (22 ± 2˚C), and were maintained under conditions of 12 hours light: 12 hours dark, with lights on at 06:00 and off at 18:00. The experimental protocol was approved by the Animal Ethics Committee in accordance with the Guidelines for the Care and Use of Laboratory Animals prepared by Tarbiat Modares University.

Cro and Crt were extracted and purified from the red dried stigmas of saffron (Crocus sativus L.), as described previously (19).

Tumors were induced in the rats using NMU (Sigma, St. Louis, MO) injection using the method explained previously (9). Briefly, NMU was dissolved in physiologic saline and then adjusted to pH=4.0 with acetic acid for activation. NMU was administered by intraperitoneal injection within 30 minutes of preparation at a dose of 50 mg/kg body weight to the rats at 50, 65 and 80 days of age. Cro and Crt were administrated by gavage with a dose of 100 mg/kg once every three days from 30 or 57 days of age, as explained below, up to the end of the experiment, which lasted 16 weeks. Less than one week after the last gavage, animals were sacrificed under anesthesia.

The rats were randomly divided into eight groups, 10 in each group. Groups were labelled as follows:

C: Control group with no treatment.

T: Tumor induced group using NMU injection.

Cro-I/Crt-I: I for prevention at initiation stage. Rats were given crocin or crocetin by gavage when they were 30 days old, i.e. 20 days before NMU injection.

Cro-P/Crt-P: P for prevention at promotion stage. Rats were given crocin or crocetin, respectively, one week after the first NMU injection (at about 57 days old).

Cro-C/Crt-C: Control positive groups that received Cro or Crt at 30 days old.

After NMU treatment, rats were weighed and palpated for mammary tumor appearance every week. Tumor volume (TV) was estimated by the following equation:

$$\mathrm{T.V}=\frac{4}{3}\pi {\left(\mathrm{R1}\right)}^{2}\left(\mathrm{R2}\right)$$

where R1 and R2 are the tumors diameters.Other estimated parameters were: latency period (LP), which is the number of days between the first NMU injection and the appearance of the first tumor; tumor incidence (TI), which is the percentage of rats that developed at least one tumor; and the mean tumor number per rat (TN), which is the number of tumors per rat in those animals developing at least one tumor.

Four months (120 days) after NMU administration, the study was terminated, and mammary tumors were prepared for histological studies as described previously (9).

### Statistical analysis

To analyze differences between the data obtained in the control group and the animals with mammary tumors, the independent-sample test was applied using SPSS 16.0. All comparisons with P values below 0.05 were considered as significant.

## Results

**Fig.1:**
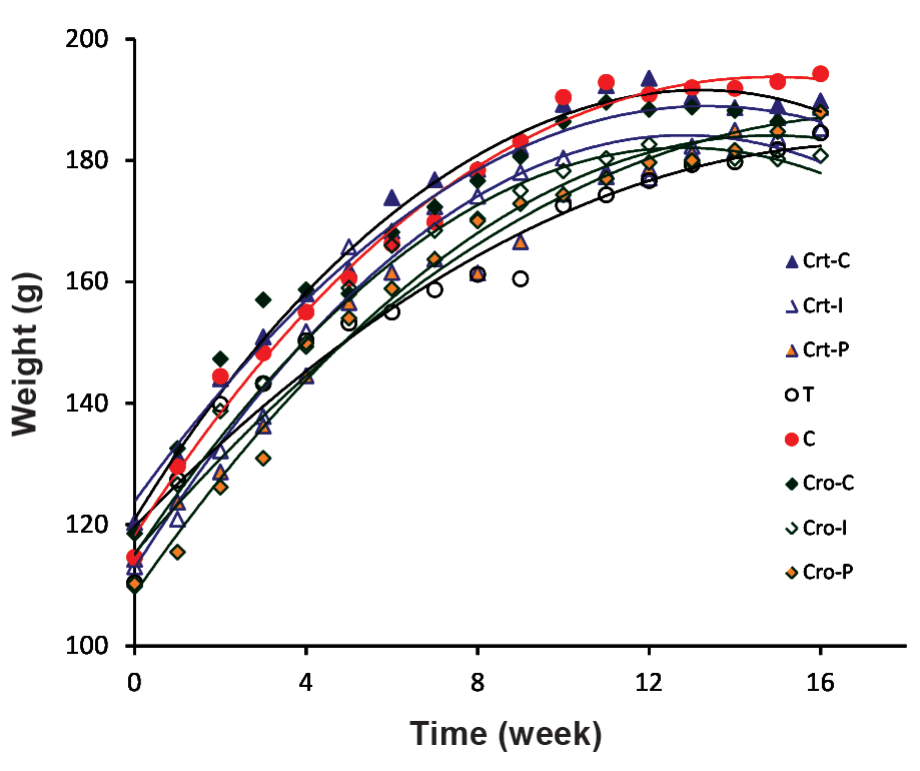
Weights of all rat groups at different weeks of the experiment. C; Control, T; NMU-treated rats, Cro; Crocin, Crt; Crocetin, I; Initiation, and P; Promotion.

Figure 1 shows alterations in the weight of all groups of rats during the experimental period. There are no significant differences in the body weight of animals in the prevention groups compared with the control groups.

No tumor was observed in the control groups that receive Cro or Crt. Thus, no data is presented for these groups in the following sections.

**Fig.2:**
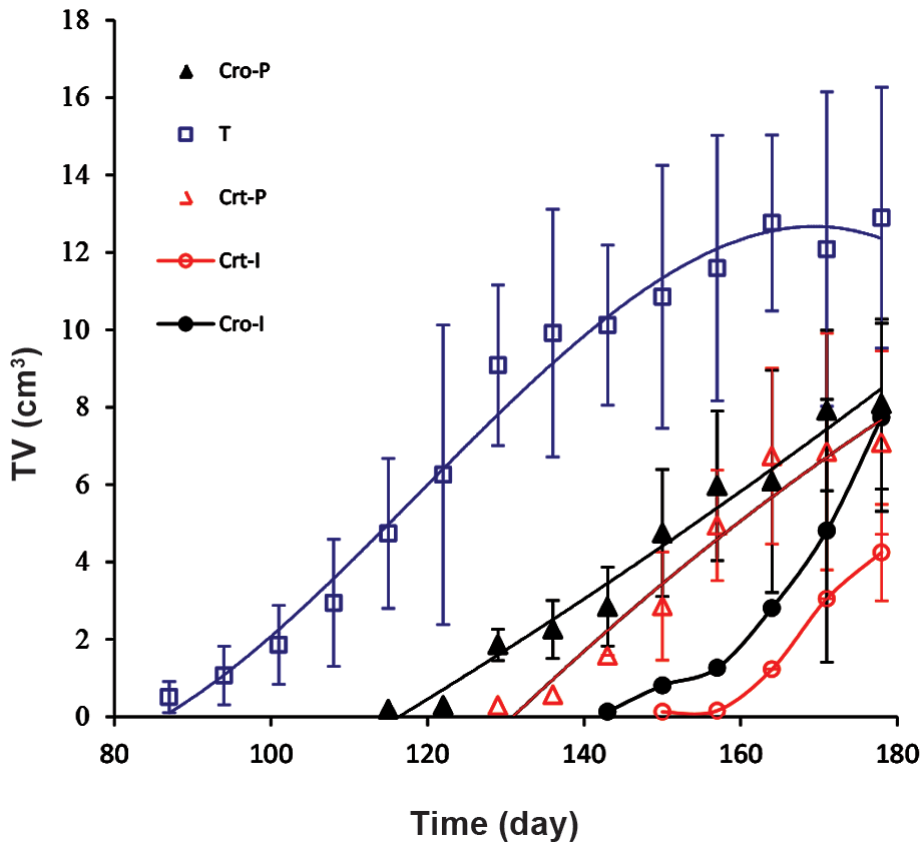
Tumor volume (TV) in each group of rats at different days of experiment. The groups name were used as defined in Figure 1. T; NMU-treated rats, Cro; Crocin, Crt; Crocetin, I; Initiation, and P; Promotion.

Figure 2 shows the results for TV in each group at successive weeks of the experiment. The results show significant changes between the groups that received Cro or Crt for prevention, either at the initiation or promotion stages, in comparison with the group that received NMU only.

**Fig.3:**
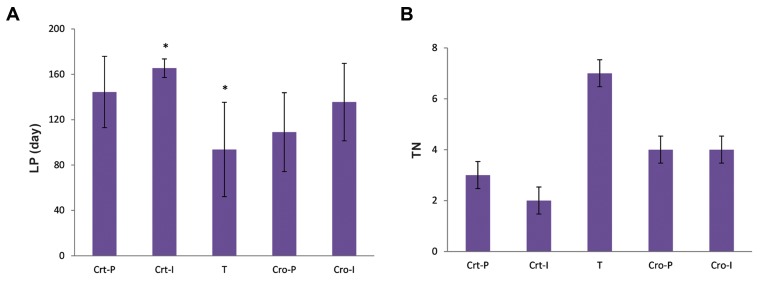
Effect of Cro and Crt on tumor parameters. A. The effect of Cro and Crt on the latency period (LP) in comparison with the rats that received NMU only and B. Tumor number (TN) in each group of rats. The groups name were used as defined in Figure 1. T; NMU-treated rats, Cro; Crocin, Crt; Crocetin, I; Initiation, P; Promotion, and *; The significant changes between the group T in compari- son with the other groups, P<0.05.

Figure 3A shows the effect of Cro and Crt on the LP in comparison with rats that were given NMU only. Figure 3B represents the TN in each group. TI % was 77% in group T, but only 22% in the Crt-I and 44% in the Cro-I groups (average 33% at initiation stage); and 42% in the Crt-P and 57% in the Cro-P groups (average 49% at promotion stage). All prevention groups showed statistically significant reductions in TN and TI compared to the control group.

**Fig.4:**
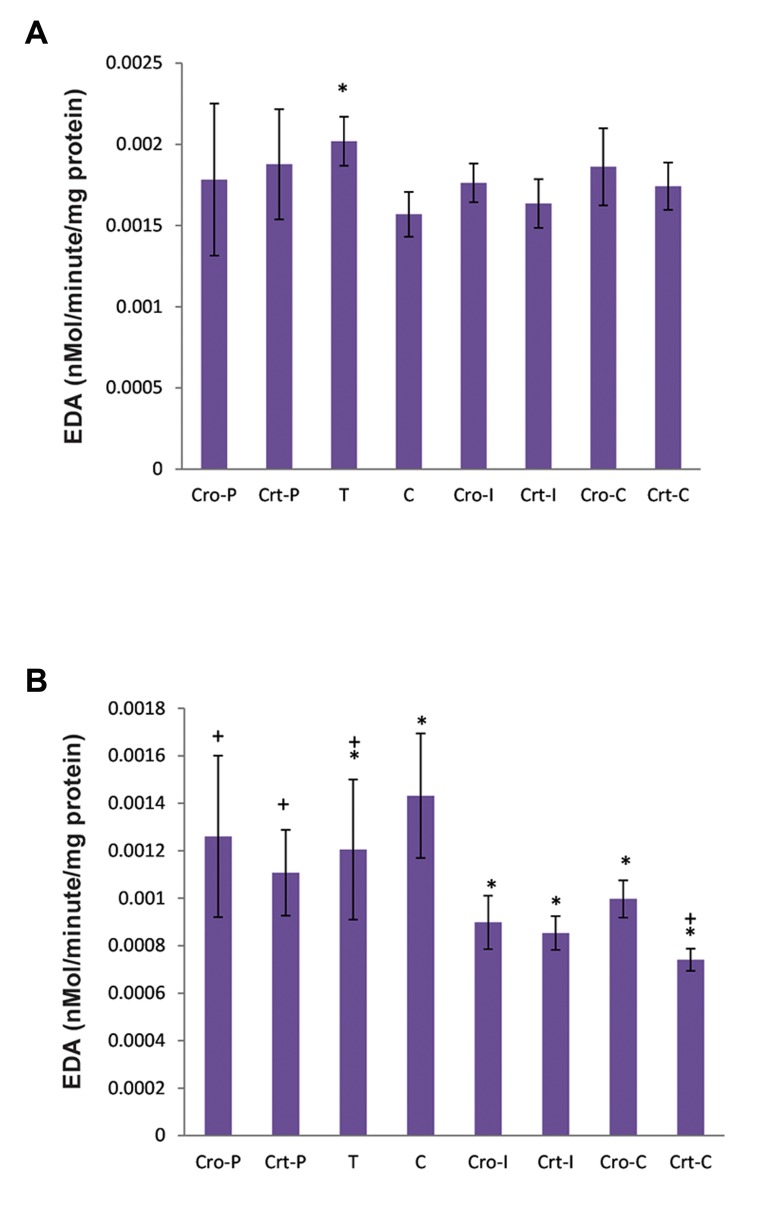
EDA activities in the A. Brain and B. Ovary of rats at the end of experiment. The groups name were used as defined in Figure 1. EDA specific activity was defined as nano-mole per minutes per mg of protein. EDA; Enkephaline degrading aminopeptidase, C; Control, T; NMU-treated rats, Cro; Crocin, Crt; Crocetin, I; Initiation, P; Promotion, *; The significant differences between the control group (C) in comparison with the other groups, P<0.05, and +; The significant differences between the group T with other groups, P<0.05.

Alterations in the brain (A) and ovary (B) EDA activities are shown in Figure 4. EDA activity was significantly increased in the brain of rats in the T group. However, there were no significant changes in enzyme activity in the intervention groups, either Cro or Crt. In contrast, EDA activities of the ovary were decreased in the T group and prevention groups in comparison with the control group.

## Discussion

This study examined the preventive effect of Cro and Crt on NMU-induced breast cancer at both the initiation and promotion stages. The results indicated the effectiveness of saffron carotenoids in the prevention of chemically induced carcinogenesis in the rat. All parameters, like TV, LP, TN and TI%, were lower in the NMU-exposure groups treated with saffron carotenoids compared with the untreated group. This means that both Cro and Crt are effective in the prevention of tumor induction, but Crt is the more effective agent. In addition, prevention at the initiation stage is more effective than at the promotion stage.

The role of saffron carotenoids (Cro and Crt) as cancer therapeutics has been extensively reviewed by our group (13, 15). According to our literature survey, the first preventive study using saffron was reported in 1991. In that report, topical application of saffron extract resulted in the inhibition of skin cancer induced by [7,12-dimethylbenz[a] anthracene (DMBA)/croton oil] in mice both at the initiation and promotion stage. The saffron extract, acting as an inhibitor of chemical-induced soft tissue sarcomas, has also been found to reduce papillomas in albino mice (20). Subsequent to that report, the chemopreventive effect of saffron extract in different types of cancers has been documented (21). The antioxidant activity of phytochemicals, including saffron extract, is now considered an important mechanism of chemoprevention in different tissues (22). The antioxidant activity of crocin, as a saffron carotenoid, has also been determined and a simple method based on this property introduced for evaluation of the antioxidant activity of biological samples (23, 24).

In addition, the beneficial effect of saffron carotenoids on increasing the activity of the antioxidant defense system has been reported (25, 26), and may provide an alternate method of protecting the organism against a carcinogen. Previous studies by our group and other research groups have also shown the chemotherapeutic effect of both Cro and Crt in different cancers (4, 18, 27). The exact molecular mechanism by which the saffron carotenoids exert their anticancer properties is not yet known, but they may induce apoptosis in tumor cells through different mechanisms, including changes in the Bax/Bcl-2 ratio and activation of caspases (4, 18). They can also induce cell cycle arrest through alteration in cylcin D1, p21 and p53 (28). Cro can also inhibit telomerase in HepG2 cells (29). In addition, both Cro and Crt interact with various DNA sequences, including telomeric DNA structures, but Crt-DNA complexes are formed at lower concentrations of Crt, making it a more potent component than Cro (30, 31).

As mentioned in the results, the concentrations of Cro and Crt used in our study were the same as those effective in the treatment of cancer (18, 28). Among these two components, Crt (with 22 and 42% TI in the initiation and promotion stages, respectively) was more effective than Cro (with 44 and 57% TI in the initiation and promotion stages, respectively) in cancer prevention (TI=77% in T group without any other intervention). The intervention in the initiation stage (average 33%) was also more effective than the intervention in the promotion stage (average 45%).

A structural study indicated that Crt has two carboxyl residues at the two ends of the carotenoid backbone, but Cro terminates in two gentiobiosyl or glycosyl residues at each end (32). According to our literature survey, the mechanism for their entrance into the cell has not yet been reported. Because of the hydrophobic structure of Crt, it may pass through the cell membrane, enter the cell and exert its biological effect. However, the presence of two hydrophilic groups in Cro may interfere with its penetration into the cell and its diffusion across hydrophobic domains of biological membranes.

Various pharmacokinetic studies have indicated that Cro cannot be absorbed readily from the gastrointestinal tract and needs to be converted to Crt (33-35). Some Cro was detected in the feces of rats after oral administration (35), while some was converted to Crt and was detected in the serum (33, 34). Two hours after oral administration of saffron Crt was also detected in the serum of volunteers (36). These findings support the results obtained in the present study in which the oral administration of Crt, by gavage, was more effective than Cro in cancer prevention. We also recently showed the effectiveness of orally administered saffron aqueous extract and Cro (to a lesser degree in comparison with saffron extract) in the prevention of metabolic syndrome in patients with Schizophrenia (37). Since toxicity studies have indicated the nontoxic nature of saffron and its carotenoids at the therapeutic doses (38-40), application of these components in humans to prevent cancer is recommended.

Results indicated increases in the body weight of the rats during the study. Although the rats in groups C and T had the highest and the lowest average body weight, respectively, but no statistically significant differences were observed. There were also no significant changes between the weights of different organs (liver, kidney, etc.) of rats in the different groups in the present study (data not shown).

EDA specific activity in the brain and ovary of all rats in the study was also determined. It has been previously shown that EDA activity was decreased in the hypothalamus, anterior and posterior pituitary, thyroid and ovary of rats due to NMU-induced breast cancer (11). This decrease was attributed to the increased levels of ENK in all these locations. Our results also indicated a significant decrease in EDA specific activity in the ovaries of the rats due to cancer induction; although a significant increase was observed in the EDA specific activity of the brain. It is possibly due to the determination of EDA specific activity in the whole brain tissue. As the results showed, Cro or Crt treatment had no significant effect on the brain enzyme of control and tumoric groups, although these treatments significantly decreased ovarian EDA specific activity, even in the control groups. This means that Cro/Crt prevented the abnormal changes of this enzyme in the brain, but may cause an increase in ENK in ovarian tissue. Since EDA activity is influenced by hormonal status (estrogen/progesterone) in mouse (41), it seems that the interpretation of the results obtained here requires further investigation in the near future.

## Conclusion

Both Cro and Crt decreased the induction of NMU-induced breast cancer tumors in female rat. All parameters such as TV, LP, TI and TN were significantly decreased after treatment with Cro and Crt at both the initiation and promotion stages. However Crt was a more effective chemopreventive agent at both stages than Cro. Since prevention at the initiation stage was more effective, Cro/Crt treatment can be a good candidate for cancer prevention in people who are at risk of breast cancer.
